# Recalibration of the NBS Glass Standards of Spectral Transmittance^1^

**DOI:** 10.6028/jres.067A.056

**Published:** 1963-12-01

**Authors:** Harry J. Keegan, John C. Schleter, Marion A. Belknap

## Abstract

In 1934, Gibson, Walker, and Brown developed sets of four colored glass filters to serve as working standards of spectral transmittance for checking the reliability of spectrophotometers. Several sets of these glasses were measured carefully and reserved and designated as future reference standards. Duplicate standards evaluated by comparison with the reference standards are available by purchase to the public. The current set of reference standards was established in the years 1945 to 1947, and one of these reference standards (selenium-red) was recalibrated in 1952. This paper reports a recalibration, made in 1961 and 1962, of all four glasses (selenium-red, carbon-yellow, copper-green, cobalt-blue) on three spectrophotometers (Cary 14, Beckman DU, König-Martens). Except for the cobalt-blue standard, the values of spectral transmittance found differ from those previously assigned by amounts differing at some wavelengths by as much as or slightly more than the uncertainties estimated for the present values, though not by amounts exceeding the combined uncertainties of the present and previous determinations. The indicated changes for these three standards are fairly regular, however, and support the view that the selenium-red and carbon-yellow standards are changing chiefly by formation of a reflectance-reducing film on the surfaces. The indicated rate of upward drift is slow, and suggests that it takes about 10 years for the drift to exceed the assigned uncertainity.

## 1. Introduction

**Figure 1:**
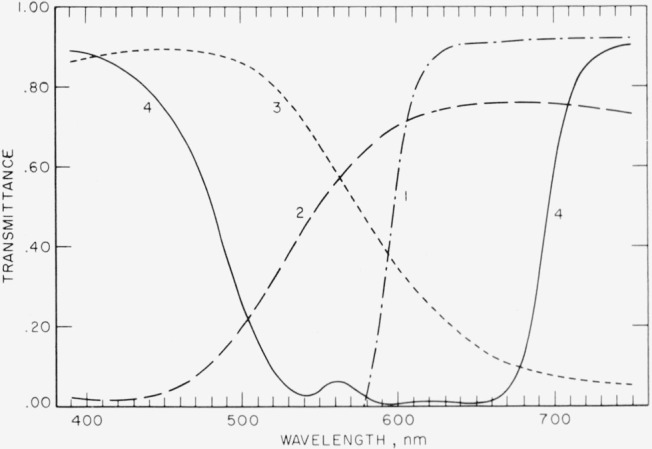
Typical spectral transmittance curves of glass duplicate standards issued by the NBS for checking the reliability of spectrophotometers. The four types are: 1. Selenium-red, 2. Carbon-yellow, 3. Copper-green, and 4. Cobalt-blue.

In 1934, Gibson, Walker, and Brown [1]^2^ described a set of four colored glass filters which they had developed to serve as standards of spectral transmittance to check the reliability of spectrophotometers. Several sets of these glasses were carefully measured at a number of wavelengths by using mercury, helium and incandescent sources and were reserved and designated as future reference standards. Duplicate standards evaluated by comparison with the reference standards were made available to the public by purchase. Although nearly nonselective filters are best suited to the detection of errors in the photometric scale, the filters chosen have spectral transmittances that vary markedly with wavelength and thus permit detection of slit-width errors and stray-energy errors as well as photometric-scale errors; thus a single selective filter may afford checks of the photometric scale over a large fraction of the whole scale [2, 3]. Table 1 identifies the glasses that have served as reference standards, and figure 1 shows the approximate spectral transmittances of these filters. The reports issued with the sets of duplicate standards gave the assigned values of transmittance at about 20 wavelengths between 390 and 750 nm 3 together with the estimated uncertainties and the temperature coefficients.

Table 2 gives for each wavelength the light source used and the estimated uncertainties and a multiple of the temperature coefficients of the reference standards expressed on the absorbance scale. Absorbance is defined as the logarithm of the reciprocal of the internal transmittance *T_i_.* Since internal transmittance for these glasses is closely equal to transmittance *T* multiplied by a constant (about 1/0.9) to correct for reflection losses [8], differences in absorbance are closely equal to differences in the logarithm of the reciprocal of transmittance *T* uncorrected for reflection losses. Accordingly the uncertainties *U_a_* in absorbance entered in table 2 are computed from the uncertainties *U* in transmittance as :

$$U_{a}=\left[\log_{10}\left(T+U\right)-\log_{10}\left(T-U\right)\right]/2$$

Similarly, the temperature coefficients *δ* of absorbance are computed from the relation :

$$-\log_{10}T_{\theta }=-\log_{10}T_{25}+b\delta \left(\theta -25\right).$$

where *θ* is the temperature of the glass in degrees C, *b* is the thickness of the glass in mm, and −log_10_*T*_25_ is the absorbance measurement made at 25 °C. The temperature coefficient *δ* is thus the change in −log_10_*T* (closely equal to the change in −log_10_*T_i_*) for a temperature change of 1 °C, and a thickness of 1 mm. These glass reference standards are measured at 25 °C and are used over a temperature range of ± 15 °C. For convenience, because the temperature coefficient is relatively small compared with the value of absorbance, a quantity *d* equal to 15 times the temperature coefficient is used to designate the change in absorbance for a temperature change of 15 °C, as follows:

$$-\log_{10}T_{\theta }=-\log_{10}T_{25}+bd\left(\theta -25\right)/15.$$

This relationship is considered valid for values of *θ* between 0 and 50 °C.

The current set of reference standards was measured in the years 1945 to 1947. A detailed analysis of the data obtained for one of the reference standards (cobalt-blue, Corning G 55A *β*^9^) was published by Gibson and Balcom [4] in 1947. The uncertainties, *U*, were estimated as the huge error (4.9 times the probable error) of the mean values of transmittance. In 1950, Gibson and Belknap [5] published a study of the permanence of samples of the carbon-yellow, the copper-green, and the cobalt-blue glasses exposed under clear glass to south skylight and sunlight for periods up to three years. Expressed in terms of transmittance change Δ*T*, these samples were found to change but slightly, excepting only the copper-green glass below 500 nm, where there appears to be a definite solarization effect.

No such study was made for ordinary use of these glasses in the laboratory. It was assumed that if the glasses would change only slowly with extensive exposure to sunlight under glass, they would not change significantly in many years of ordinary use. A redetermination of the spectral transmittance of the current reference standard for selenium-red glass was carried out in 1952 and resulted in slight changes in the assigned values, but it could not be determined with certainty that the transmittance had changed. The 1946 and 1952 data are given in table 3.

Meanwhile, interest in the duplicate standards was increasing. Table 4 shows the number of duplicate standards of each type of glass issued in the years 1933–1942, 1943–1946, and 1947–1962.

A set of these glass standards, issued in 1958 to the Frankford Arsenal, was submitted in October 1961 for recalibration. A routine comparison of them with the same reference standards used in the original calibration showed that the transmittances for each of the four glasses bore a significantly different relation to the corresponding reference standard than in 1958. It was presumed that this changed relation referred primarily to the duplicate standards, whose uses and possible exposure to radiant energy at the Frankford Arsenal were unspecified, rather than to the reference standards known to be irradiated only by incandescent-lamp light for short periods of time at infrequent intervals; and this presumption was subsequently proved to be correct. Nevertheless, check data obtained in 1952, 1953, 1959, and 1960 for the reference standards were reviewed. Although no single set of data proved that any of the reference standards had certainly changed, all four sets of data considered together showed some evidence of a slow continuing drift in spectral transmittance of the selenium-red, the carbon-yellow and the copper-green reference standards. The cobalt-blue glass alone showed no change in spectral transmittance. An extensive recalibration of all four reference standards was then undertaken. It is the purpose of the present paper to describe this recalibration, and to discuss its results.

## 2. Method of Spectrophotometry

The recalibration of the four reference standards of spectral transmittance was carried out by means of the Cary 14 recording spectrophotometer, the Beckman DU manual spectrophotometer [6], and the König-Martens visual spectrophotometer [7]. Table 5 lists the spectrophotometers used in the several calibrations of reference standards of spectral transmittance. The procedural details of the present recalibration are given below.

### 2.1. Cary Recording Spectrophotometer

Two sets of measurements of each of the four reference standards of spectral transmittance were made on the Cary recording photoelectric spectrophotometer (Model 14M, Serial No. 173) by means of the absorbance (−log*T*) scale. At the same time measurements were made of a clear borosilicate crown glass, 1.0 mm thick, of known index of refraction and Abbe value (*n*_D_= 1.517, *v*=64.5).^4^ This index and Abbe value are the same as those found for the borosilicate crown glass, 1.0 mm thick, whose spectral transmittances were known from a previous study [8]. These known spectral transmittances were used as a photometric-scale correction for transmittances of the reference standards greater than 40 percent. For each wavelength the ratio *R*_λ_ of the reference-standard transmittance to that of the borosilicate crown glass was computed from the absorbance readings. The value *T*_λ_ of the transmittance of the reference standard was computed as:

$$T_{\lambda }=R_{\lambda }T_{0\lambda },$$

where *T*_0λ_ is the known spectral transmittance of the borosilicate crown glass [8]. For *T*_λ_ less than 40 percent the value corresponding to the reading of the absorbance scale was accepted without correction. The wavelength calibration of the instrument was carried out as described by Keegan, Schleter, and Judd [9]. All measurements refer to the specimen at 25 °C.

### 2.2. Beckman DU Spectrophotometer

The spectral transmittances of the reference standards were measured on the Beckman DU (“BQ-1”) spectrophotometer with incandescent source after calibration of wavelength scale at emission lines of mercury, helium, hydrogen, neon, and cesium. Frequent checks of the wavelength calibration were made by means of the mercury line at 546.1 nm. Each standard was measured in two positions in the sample holder for each set of readings. The holder was positioned so as to place the standard near the exit slit and away from the phototube. As recommended by Gibson and Balcom [4], the measurements were made with no lens over the exit slit. The spectral transmittance of each standard was measured at selected wavelengths by ratio to a blank beam set to 100 percent transmittance as a reference. The shift from the blue-sensitive to red-sensitive phototube was made at 620 nm. The selector switch was shifted to 0.1 for transmittance readings below 10 percent to give “times-10” readings at narrower slitwidths. A purple stray-energy filter was used for all measurements taken at 390 nm. The sample compartment was surrounded by a water jacket maintained at 25 °C by a constant-temperature water bath. The numbers of sets of readings made on the reference standards are as follows:

**Table:** 

Selenium-red		Carbon-yellow	Copper-green	Cobalt-blue
				
Below	Above	Full	Full	Full
600nm	600nm	spectra	spectra	spectra
2	3	7	3	2

The extra set of readings above 600 nm for the selenium-red standard was obtained by ratio of readings for the standard to those for a clear glass (Corning 9700).

### 2.3. König-Martens Spectrophotometer

The spectral transmittances of the four reference standards were measured on the König-Martens visual spectrophotometer [7] by using emission lines of a mercury source and a helium source and, at some wavelengths, by using an incandescent source. As explained on pages 464–465 of reference [5], it was early found advisable, because of the low luminosities of the Hg and He sources at 471.3, 491.6, 667.8, and 706.5 nm, to use the incandescent source at these wavelength settings, and this has been done in all later work on the König-Martens visual spectrophotometer. However, for the present standardization of the NBS reference standards, measurements were made with both incandescent and line sources at these four wavelengths. The wavelength scale was checked by settings on certain of the mercury and helium lines each time the instrument was used and each time the width of the ocular slit was changed. The widths of the collimator and ocular slits were 0.2 mm for the readings taken by means of the incandescent source, and were varied from 0.2 to 0.4, or 0.5 mm for the line sources. Accurately calibrated sector disks (transmittances approximately 1, 10, 50, and 60 percent) were used for measurement of low transmittances or for transmittances near to those of the sectors to increase the accuracy of the readings. Stray-energy filters were used at wavelengths near the two ends of the visible spectrum and at wavelengths of low transmittance. Each set of readings consisted of 20 readings of angle on the Martens photometer; first, 10 for the standard inserted in one beam, and second, 10 for it in the other beam. The transmittance was computed as the cotangent of the first angle multiplied by the tangent of the second. At least two sets of readings were taken for each standard at each selected wavelength between 404.7 and 660 nm and a few additional readings between 660 and 706.5 nm because of the added uncertainty of the settings in this region of low luminosity. All readings were made with the standards in a holder maintained at 25° C by means of a constant-temperature water bath.

## 3. Reduction of Data and Estimates of Uncertainty

The spectral transmittances assigned to the reference standards are weighted means of the transmittances found individually by the three spectrophotometers used. The weights assigned to the individual values of transmittance for a particular standard at a particular wavelength were based on the known relative capabilities of the three instruments. For example, the values obtained by means of the König-Martens visual spectrophotometer in spectral regions yielding observing fields of low luminance (such as near the extremes of the visible spectrum) were given less weight because of the resulting unreliability of the visual settings. On the other hand values of spectral transmittance obtained by means of an emission line in a spectral region of rapid variation of transmittance with wavelength were given more weight.

In tables 6, 7, 8, and 9, are given for each of the four reference standards and for each of the three instruments at the respective wavelengths, the average value of the transmittance so measured, the assigned weight, the range in transmittance, the adopted weighted mean, and the estimated uncertainty in the value of transmittance.

The year or years of measurement on the indicated spectrophotometers are shown in the parenthesis under the name of the instrument. The uncertainty of the assigned value of transmittance at a particular wavelength was made the same as that assigned previously (see table 3); with exceptional cases where it was agreed that a change was necessary. In these cases the uncertainty was estimated from the range of the individual values obtained on the three instruments averaged over a spectral region variable in extent from 0 to 50 nm and centered on that wavelength. Some smoothing of these values was resorted to whenever no reason was apparent for a rapid change in uncertainty with wavelength. In no case was the uncertainty allowed to be less than one-half the range of the spectrophotometric data. These ranges correspond roughly to estimates of three times the standard deviation of the adopted value [10].

In table 10 the change in transmittance between the present value and the previously adopted value of spectral transmittance is given for each of the four reference standards at the respective wavelengths.

**Figure 2:**
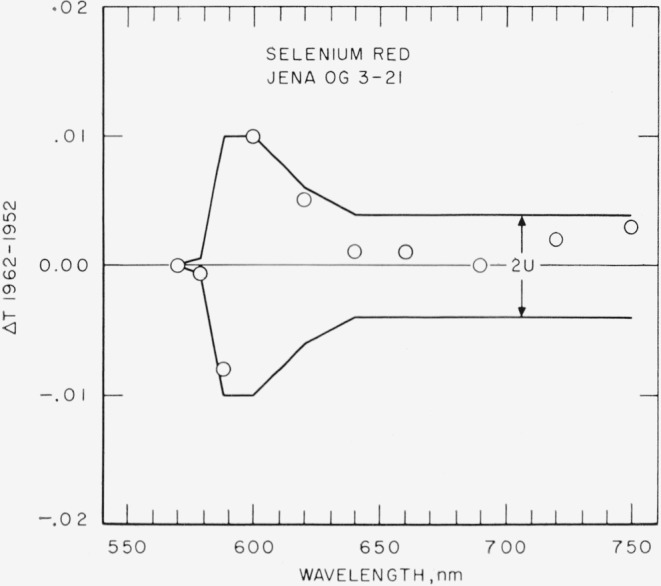
Selenium-red reference standard. Differences (shown as circles) between spectral transmittances assigned in 1962 and in 1952 compared to estimates (shown as broken lines) of the uncertainty of the 1962 recalibration.

**Figure 3:**
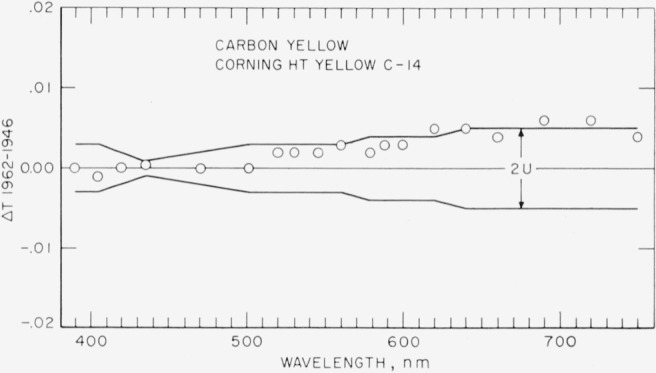
Carbon-yellow reference standard. Differences (shown as circles) between spectral transmittances assigned in 1962 and in 1946 compared to estimates (shown as broken lines) of the uncertainty of the 1962 recalibration.

**Figure 4:**
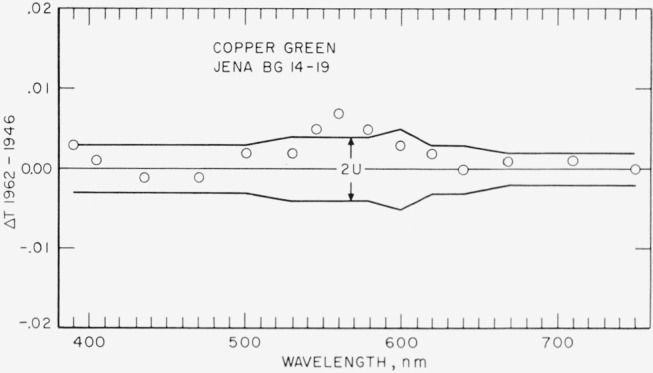
Copper-green reference standard. Differences (shown as circles) between spectral transmittances assigned in 1962 and in 1946 compared to estimates (shown as broken lines) of the uncertainty of the 1962 recalibration.

**Figure 5:**
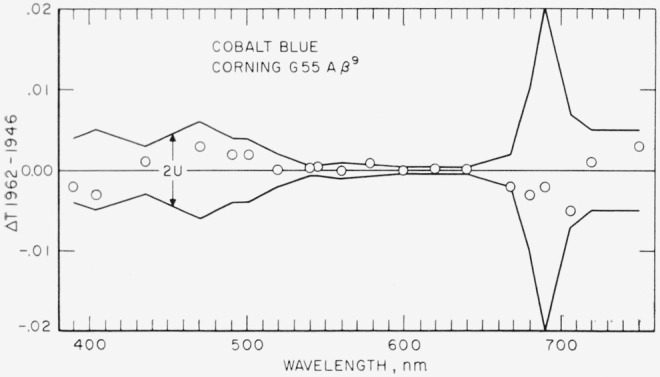
Cobalt-blue reference standard. Differences (shown as circles) between spectral transmittances assigned in 1962 and in 1946 compared to estimates (shown as broken lines) of the uncertainty of the 1962 recalibration.

Figures 2, 3, 4, and 5 show as circles these differences in spectral transmittance assigned by the present recalibration and that assigned by the previous calibration for the selenium-red, the carbon-yellow, the copper-green, and the cobalt-blue standards. They also show as broken lines centered about the abscissa the estimates of the uncertainties of the values of spectral transmittance resulting from the present recalibration.

## 4. Discussion

It will be noted from figure 2 that three of the ten newly assigned values of spectral transmittance for the selenium-red reference standard differ from the previous assigned values by amounts approaching the estimated uncertainty. Because the previously assigned values are uncertain by about the same amount, this recalibration by itself fails to prove that the selenium-red standard has changed since 1952. The results of the two spectrophotometers (König-Martens and Beckman DU “BQ-1”) common to the 1952 determination and the present determination, however, both indicate that a change has occurred. Furthermore, comparison of the 1947 with the 1952 determination shows similar changes. It seems likely that indications of increases in transmittance between 620 and 750 nm correspond to formation of a reflectance-reducing film on the surfaces. Several of the duplicates of this reference standard show visible evidence of such films. The decreases in transmittance indicated near the cut-off of the transmittance curve may not be real. (See fig. 2, wavelengths 570 and 587.6 nm). In any case, there is no established cause for such a change.

Figure 3 gives an indication of formation of a reflectance-reducing film on the surfaces of the carbon-yellow reference standard similar to that by figure 2 for the selenium-red standard. Note that the indicated discrepancies between the present and the previous assignments of spectral transmittance are roughly proportional to the spectral transmittances themselves, (see fig. 1), as would be expected from the formation of a reflectance-reducing film.

Figure 4 indicates some increases (390, 405, 500–620 nm) and some decreases (436, 471 nm) in assigned values of spectral transmittance. This indicated pattern is not consistent with the hypothesis that a reflectance-reducing film has been forming on the copper-green reference standard. If the indicated changes are real, and this has not been proved, they must be ascribed to a cause other than film formation (such as a slow chemical change in the glass), or to a combination of film formation with other cause or causes unknown.

Figure 5 gives no plausible suggestion that the cobalt-blue reference standard is changing even slowly. In table 10 the indicated changes are either small compared to the estimated uncertainty of the present determinations, or irregular.

In summary, it may be stated that three of the reference standards of spectral transmittance are probably subject to a slight impermanence, the maximum changes in a 10-year period being of the order of magnitude of the uncertainties in their calibration. Owners of duplicate standards not subjected to unusually severe usage (exposure to high-energy particles, ultraviolet energy, high temperatures, or chemical fumes) may find in figures 2 to 5 a reasonably valid basis for revising the adopted transmittance for their duplicate standards. In the absence of information to the contrary, the changes may be assumed as a first approximation to be proportional with time, and the time for recently issued duplicates (1952 to 1961) should be counted from 1952 for the selenium-red standards, and from 1946 for the carbon-yellow, the copper-green, and the cobalt-blue standards.

## Figures and Tables

**Table 1:** Identification of the glasses used as reference standards

No.	Designation	1934–1940	1945–1947
			
1	Selenium-red	Jena OG 3–8	Jena OG 3–21
		Jena OG 3–10	
2	Carbon-yellow	Corning	Corning
		HT Yellow *α*^16^	HT Yellow C-14
		HT Yellow *α*^7^	
3	Copper-green	Jena BG 14–1	Jena BG 14–19
		Jena BG 14–10	
4	Cobalt-blue	Corning	Coming
		G 55A *α*^11^	G 55A *β*^9^
		G 55A *α*^3^	

**Table 2:** Estimated uncertainty in the value of absorbance, *U*_a_, and the change d, in absorbance for temperature change of 15° C, for the selected wavelengths for incandescent (Inc.) and line (*Hg* and *He*) sources used for the four reference standards

Wavelength	Source	Selenium-red		Carbon-yellow		Copper-green		Cobalt-blue	
									
λ *in nm*		*U_a_*	*d*	*U_a_*	*d*	*U_a_*	*d*	*U_a_*	*d*
390	Inc	……	……	±0.052	−0.0005	±0.002	0.000	±0.002	0.000
404.7	Hg	……	……	.069	−.0025	.002	.000	.002	.000
420	Inc	……	……	.046	−.0025	……	……	……	……
435.8	Hg	……	……	.014	−.0015	.001	.000	.002	.000
471.3	He	……	……	.011	+.001	.001	.000	.004	.000
491.6	Hg	……	……	……	……	……	……	.005	+.0005
501.6	He	……	……	.006	+.0035	.002	.000	.007	+.001
520	Inc	……	……	.004	+.0035	……	……	.010	+.0005
530	Inc	……	……	.003	+.003	.002	.000	……	……
540	Inc	……	……	……	……	……	……	.008	−.003
546.1	Hg	……	……	.003	+.0025	.003	.000	.008	−.004
560	Inc	……	……	.002	+.002	.003	.000	.007	−.003
570	Inc	0.000	……	……	……	……	……	……	……
578	Hg	±.06	+0.17	.003	+.0015	.004	−.0005	.008	−.004
587.6	He	.04	+.11	.003	+.0015	……	……	……	……
600	Inc	.008	+.022	.002	+.001	.006	−.0005	.024	−.005
620	Inc	.003	+.002	.002	+.0005	.005	−.001	.017	–.003
640	Inc	.002	.000	.003	+.0005	.007	−.001	.023	–.005
660	Inc	.002	.000	.003	.000	……	……	……	……
667.8	He	……	……	……	……	.007	−.001	.027	+.004
680	Inc	……	……	……	……	……	……	.033	+.006
690	Inc	.002	.000	.003	.000	……	……	.026	+.004
706.5	He	……	……	……	……	……	……	.004	+.002
710	Inc	……	……	……	……	.012	−.001	……	……
720	Inc	.002	.000	.003	−.0005	……	……	.003	+.0005
750	Inc	.002	.000	.003	−.0005	.015	−.0005	002	.000

**Table 3:** Previously adopted value of transmittance, *T*, and estimated uncertainty in the values of transmittance, *U*, at the respective wavelengths for the four reference standards

Wavelength	Selenium-red (1952)*		Carbon-yellow (1946)*		Copper-green (1946)*		Cobalt-blue (1946)*	
								
λ *in nm*	*T*	*U*	*T*	*U*	*T*	*U*	*T*	*U*
390	……	……	0.025	±0.003	0.862	±0.003	0.895	±0.004
404.7	……	……	.020	.003	.877	.003	.884	.005
420	……	……	.019	.002	……	……	……	……
435.8	……	……	.0240	.0007	.893	.004	.806	.003
471.3	……	……	.081	.002	.894	.004	.612	.006
491.6	……	……	……	……	……	……	.344	.004
501.6	……	……	.208	.003	.859	.005	.245	.004
520	……	……	.316	.003	……	……	.091	.002
530	……	……	.379	.003	.760	.004	……	……
540	……	……	……	……	……	……	.0308	.0006
546.1	……	……	.479	.003	.671	.004	.0335	.0006
560	……	……	.557	.003	.585	.004	.064	.001
570	0.000	……	……	……	……	……	……	……
578	.0042	±0.0005	.636	.003	.473	.004	.0273	.0005
587.6	.118	.01	.668	.003	……	……	……	……
600	.550	.02	.699	.003	.350	.003	.0074	.0002
620	.852	.007	.731	.004	.256	.003	.0100	.0002
640	.904	.005	.747	.005	.187	.003	.0074	.0002
660	.914	.005	.754	.005	……	……	……	……
667.8	……	……	……	……	.122	.002	.034	.002
680	……	……	……	……	……	……	.14	.01
690	.919	.005	.755	.005	……	……	.34	.02
706.5	……	……	……	……	……	……	.713	.007
710	……	……	……	……	.074	.002	……	……
720	.918	.005	.748	.005	……	……	.845	.005
750	.917	.005	.730	.005	.057	.002	.901	.005

* Date of adoption of values.

**Table 4:** Numbers of duplicate standards issued

Years	Calibration of duplicate standards in charge of:	Selenium-red	Carbon-yellow	Copper-green	Cobalt-blue	Total
						
1933–1942	Mabel E. Brown and Geraldine W. Haupt.	7	20	10	15	52
1943–1946	Margaret M. Balcom and Lois A. Peterson.	0	11	7	16	34
1947–1962	Marion A. Belknap	20	161	113	99	393
						
	Totals	27	192	130	130	479

**Table 5:** Spectrophotometers used in the several calibrations of reference standards of spectral transmittance

Spectrophotometer	Date of calibration
	
König-Martens visual	1930–1940 1940–1950 1950–1960 1960–
Gibson photoelectric and thermoelectric.	1930–1940 1940–1950
Hilger photographic sector-photometer	1930–1940
Beckman DU photoelectric (“BQ-1”)	1950–1960 1960–
Beckman DU photoelectric (“BQ-2”)	1940–1950
Cary 14M recording photoelectric	1960–

**Table 6:** Spectral transmittance, *T*, of selenium-red reference standard Jena OG 3–21, 1.7 mm, as measured on indicated spectrophotometers; assigned weight, *W*, range, *R*, adopted weighted mean, and estimated uncertainty of value of transmittance, *U*

Wavelength	König-Martens (1962)*		Beckman DU (1961–1962)*		Cary Model 14 (1962)*		Range	Adopted weighted mean	Estimated uncertainty
									
λ *in nm*	*T*	*W*	*T*	*W*	*T*	*W*	*R*	*T*	*U*
$\begin{array}{l} 390\\ \text{to}\\ 570 \end{array}\}$	0.000	……	0.000	……	0. 000	……	0.000	0.000	……
578	.0041	4	.0033	2	.0034	4	.0008	.0037	±0.0005
587.6	.115	4	.102	3	.110	3	.013	.110	.01
600	.564	3	.556	4	.561	3	.008	.560	.01
620	.858	4	.856	4	.861	2	.005	.857	.006
640	.906	4	.903	4	.909	2	.006	.905	.004
660	.913	4	.914	4	.920	2	.007	.915	.004
690	.917	3	.918	5	.925	2	.008	.919	.004
720	.918	2	.919	6	.926	2	.008	.920	.004
750	.919	1	.919	7	.925	2	.006	.920	.004

* Date of measurement.

**Table 7:** Spectral transmittance, *T*, of carbon-yellow reference standards Corning HT yellow C14, 2.0 mm, as measured on indicated spectrophotometers; assigned weight, *W*, range, *R*, adopted weighted mean and estimated uncertainty of transmittance, *U*

Wavelength	König-Martens (1962)*		Beckman DU (1961–1962)*		Cary Model 14 (1962)*		Range	Adopted weighted mean	Estimated uncertainty
									
λ *in nm*	*T*	*W*	*T*	*W*	*T*	*W*	*R*	*T*	*U*
390	----------	----------	0.025	5	0.025	5	0.000	0.025	±0.003
404.7	0.019	3	.019	4	.019	3	.000	.019	.003
420	----------	----------	.019	5	.019	5	.000	.019	.002
435.8	.0244	4	.0234	2	.0248	4	.0014	.0244	.0008
471.3	.081	2	.080	4	.082	4	.002	.081	.002
501.6	.209	3	.206	4	.209	3	.003	.208	.003
520	.316	3	.318	4	.320	3	.004	.318	.003
530	.381	3	.380	4	.382	3	.002	.381	.003
546.1	.480	3	.480	4	.484	3	.004	.481	.003
560	.558	3	.560	4	.562	3	.004	.560	.003
578	.634	3	.639	4	.642	3	.008	.638	.004
587.6	.671	3	.669	4	.674	3	.005	.671	.004
600	.702	4	.702	4	.704	2	.002	.702	.004
620	.736	4	.734	4	.739	2	.005	.736	.004
640	.750	4	.751	4	.756	2	.006	.752	.005
660	.756	4	.758	4	.763	2	.007	.758	.005
690	.760	3	.759	4	.764	3	.005	.761	.005
720	----------	----------	.752	6	.756	4	.004	.754	.005
750	----------	----------	.733	6	.736	4	.003	.734	.005

* Date of measurement.

**Table 8:** Spectral transmittance, *T*, of copper-green reference standard Jena BG 14–19, 2.0 mm, as measured on indicated spectrophotometers; assigned weight, *W*, range, *R*, adopted weighted mean, and estimated uncertainty of transmittance, *U*

Wavelength	König-Martens (1962)*		Beckman DU (1962)*		Cary Model 14 (1962)*		Range	Adopted weighted mean	Estimated uncertainty
									
λ *in nm*	*T*	*W*	*T*	*W*	*T*	*W*	*R*	*T*	*U*
390	……	……	0.866	6	0.864	4	0. 002	0.865	±0.003
404.7	0. 876	2	.878	4	.878	4	.002	.878	.003
435.8	.893	3	.891	4	.892	3	.002	.892	.003
471.3	.893	2	.893	4	.892	4	.001	.893	.003
501.6	.862	3	.861	4	.860	3	.002	.861	.003
530	.759	3	.765	4	.762	3	.006	.762	.004
546.1	.672	3	.678	4	.677	3	.006	.676	.004
560	.593	3	.594	4	.589	3	.005	.592	.004
578	.477	3	.481	4	.476	3	.005	.478	.004
600	.348	3	.358	4	.352	3	.010	.353	.005
620	.258	3	.257	4	.258	3	.001	.258	.003
640	.187	3	.188	4	.187	3	.001	.187	003
667.8	.123	3	.123	4	.122	3	.001	.123	002
710	.078	1	.075	5	.075	4	.003	.075	.002
750	……	……	.057	5	.057	5	.000	.057	.002

* Date of measurement.

**Table 9:** Spectral transmittance, *T*, of cobalt-blue reference standard Corning G 55 Aβ^9^, 3.0 mm, as measured on indicated spectrophotometers; assigned weight, *W*, range, *R*, adopted weighted mean, and estimated uncertainty of transmittance, *U*

Wavelength	König-Martens (1962)*		Beckman DU (1962)*		Cary Model 14 (1962)*		Range	Adopted weighted mean	Estimated uncertainty
									
λ *in nm*	*T*	*W*	*T*	*W*	*T*	*W*	*R*	*T*	*U*
390	----------	----------	0.894	6	0.892	4	0.002	0.893	±0.004
404.7	0.887	2	.880	4	.880	4	.007	.881	.005
435.8	.808	3	.808	4	.805	3	.003	.807	.003
471.3	.613	2	.618	4	.612	4	.006	.615	.006
491.6	.344	2	.351	4	.342	4	.009	.346	.004
501.6	.248	3	.249	4	.244	3	.005	.247	.004
520	.091	4	.092	3	.089	3	.003	.091	.002
540	.0314	4	.0308	3	.0310	3	.0006	.0311	.0006
546.1	.0339	4	.0334	3	.0344	3	.0010	.0339	.0006
560	.064	4	.064	3	.065	3	.001	.064	.001
578	.0290	4	.0281	3	.0274	3	.0016	.0282	.0008
600	.0073	4	.0072	3	.0073	3	.0001	.0073	.0004
620	.0100	4	.0104	3	.0104	3	.0004	.0102	.0004
640	.0075	6	.0082	2	.0074	2	.0008	.0076	.0004
667.8	.032	4	.032	4	.034	2	.002	.032	.002
680	.127	2	.132	5	. 139	3	.012	.133	.01
690	.310	2	.338	5	.352	3	.042	.337	.02
706.5	.708	2	.703	4	.714	4	.011	.708	.007
720	----------	----------	.844	7	.852	3	.008	.846	.005
750	----------	----------	.902	7	.909	3	.007	.904	.005

* Date of measurement.

**Table 10:** Change in spectral transmittance, Δ*T*, for the four reference standards between the present adopted weighted mean and the previously adopted values

Wave-length	Selenium-red	Carbon-yellow	Copper-green	Cobalt-blue
				
λ *in nm*	Δ*T* (1962–1952)	Δ*T* (1962–1946)	Δ*T* (1962–1946)	Δ*T* (1962–1946)
390	……	0.000	+0.003	−0.002
404.7	……	−.001	+.001	−.003
420	……	.000	……	……
435.8	……	+.0004	−.001	+.001
471.3	……	.000	−.001	+.003
491.6	……	……	……	+.002
501.6	……	.000	+.002	+.002
520	……	+.002	……	.000
530	……	+.002	+.002	……
540	……	……	……	+.0003
546.1	……	+.002	+.005	+.0004
560	……	+.003	+.007	.000
570	0.000	……	……	……
578	−.0005	+.002	+.005	+.0009
587.6	−.008	+.003	……	……
600	+.010	+.003	+.003	−.0001
620	+.005	+.005	+.002	+.0002
640	+.001	+.005	.000	+.0002
660	+.001	+.004	……	……
667.8	……	……	+.001	−.002
680	……	……	……	−.003
690	.000	+.006	……	−.002
706.5	……	……	……	−.005
710	……	……	+.001	……
720	+.002	+.006	……	+.001
750	+.003	+.004	.000	+.003

## Footnotes

2 Figures in brackets indicate the literature references at the end of this paper.

3 Nanometers, 10^−9^ meters, formerly called millimicrons.

4 We are indebted to Irving Malitson for these measurements of index of refraction and Abbe value
